# Phosphorylation of the Chloroplastic Metalloprotease FtsH in *Arabidopsis* Characterized by Phos-Tag SDS-PAGE

**DOI:** 10.3389/fpls.2019.01080

**Published:** 2019-09-10

**Authors:** Yusuke Kato, Wataru Sakamoto

**Affiliations:** Institute of Plant Science and Resources (IPSR), Okayama University, Kurashiki, Japan

**Keywords:** FtsH, thylakoid, chloroplast, protein phosphorylation, protease, photosynthesis

## Abstract

FtsH is an essential ATP-dependent metalloprotease for protein quality control in the thylakoid membrane of *Arabidopsis thaliana* chloroplasts. It is required for chloroplast development during leaf growth, and particularly for the specific degradation of photo-damaged D1 protein in the photosystem II (PSII) complex to maintain photosynthesis activity. In the thylakoid membrane, the reversible phosphorylation of proteins is known to control the activity and remodeling of photosynthetic complexes, and previous studies implicate that FtsH is also phosphorylated. We therefore assessed the phosphorylation status of FtsH and its possible role in the regulatory mechanism in this study. The phosphorylation level of FtsHs that compose the FtsH heterohexameric complex was investigated by phosphate-affinity gel electrophoresis using a Phos-Tag molecule. Phos-tag SDS-PAGE of thylakoid proteins and subsequent immunoblot analysis showed that both type A (FtsH1/5) and type B (FtsH2/8) subunits were separable into phosphorylated and non-phosphorylated forms. Neither different light conditions nor the lack of two major thylakoid kinases, STN7 and STN8, resulted in any clear difference in FtsH phosphorylation, suggesting that this process is independent of the light-dependent regulation of photosynthesis-related proteins. Site-directed mutagenesis of putatively phosphorylated Ser or Thr residues into Ala demonstrated that Ser-212 may play a role in FtsH stability in the thylakoid membranes. Different phosphorylation status of FtsH oligomers analyzed by two-dimensional clear-native/Phos-tag SDS-PAGE implied that phosphorylation partially affects FtsH complex formation or its stability.

## Introduction

FtsH is an ATP-dependent zinc metalloprotease with a transmembrane domain. The N-terminal transmembrane domain anchors FtsH to cellular membranes with their ATPase domain facing the membranes. Thus, FtsH protease pulls their substrates out of membranes in an ATP-dependent manner and degrades them to small peptides in the proteolytic chamber (Ito and Akiyama, 2005; Nishimura et al., 2016). FtsH protease is originally identified in a temperature-sensitive phenotype of *Escherichia coli*. FtsH protein homologues have been found in other prokaryotes as well as in organelles of bacterial origin, such as mitochondria and chloroplasts. The fundamental function of FtsH protease is the quality control of membrane proteins, but its contribution to stress responses is also suggested (reviewed by Janska et al., 2013; van Wijk, 2015; Kato and Sakamoto, 2018).

In the *Arabidopsis thaliana* genome, 12 genes encoding members of the FtsH family have been identified. Nine of these proteins (FtsH1, 2, 5, 6, 7, 8, 9, 11, and 12) are located in the chloroplast (Sakamoto et al., 2003). Additionally, the *Arabidopsis* genome encodes five proteolytically inactive homologues (FtsHi) (Wagner et al., 2012); four of them (FtsHi1, 2, 4, and 5) form a complex with Ycf2 and FtsH12 at the chloroplast inner envelope membrane (Kikuchi et al., 2018; Schreier et al., 2018). This AAA-ATPase complex associates with the chloroplast TIC complex (Nakai, 2018), and participates in the translocation of chloroplast-targeted proteins as the import motor (Kikuchi et al., 2018). Much effort has been devoted to identify and characterize the FtsH protease in the thylakoid membranes. Five FtsH homologues (FtsH1, 2, 5, 6, and 8) function in the thylakoid membrane (Sakamoto et al., 2003; Yu et al., 2004; Yu et al., 2005; Wagner et al., 2011; Zaltsman et al., 2005a). Of them, four FtsH (FtsH1, 2, 5, and 8) form a heterohexameric complex (hereafter simply called FtsH complex) in the thylakoid membrane; these homologues are divided into two types, type A (FtsH1/FtsH5) and type B (FtsH2/FtsH8) (Sakamoto et al., 2003; Yu et al., 2004; Yu et al., 2005; Zaltsman et al., 2005b). Mutants lacking FtsH5 and FtsH2 are known as *yellow variegated1* (*var1*) and *var2*; these mutants show weak and strong leaf-variegated phenotype, respectively, whereas mutants lacking FtsH1 and FtsH8 do not show the leaf-variegated phenotype (Chen et al., 2000; Takechi et al., 2000; Sakamoto et al., 2002). Both types of subunits are essential for the active FtsH complex in the thylakoid membranes and the severity of the mutant phenotype depends on the level of FtsH complex in thylakoid membranes (Chen et al., 2000; Takechi et al., 2000; Yu et al., 2004; Zaltsman et al., 2005b; Miura et al., 2007; Kato et al., 2012a). Mass spectrometry analyses suggested that *Arabidopsis* FtsH complex contains two type-A subunits and four type-B subunits in a hexameric complex (Moldavski et al., 2012), but a study in cyanobacteria (Boehm et al., 2012) and a recent research of our group (Kato et al., 2018) suggested that the ratio between type A and type B subunits in the hexameric complex is 3:3.

Another relevant phenotype which characterizes mutants lacking FtsH is photosensitivity, with higher accumulation of reactive oxygen species (ROS) in the chloroplasts due to impairment of the repairing-capacity of photosystem II (PSII) from light-induced damage (Lindahl et al., 2000; Bailey et al., 2002; Sakamoto et al., 2002; Sakamoto et al., 2003; Kato et al., 2009). Thylakoid FtsH mediates the degradation of the damaged D1 protein, which is part of the PSII reaction center (Kato et al., 2009; Kato et al., 2012b). The removal of damaged D1 protein is essential for the PSII repair cycle, which is required for the recovery of photosynthetic efficiency reduced by photoinhibition (reviewed by Nixon et al., 2010; Järvi et al., 2015). A possible regulation mechanism of FtsH activity by prohibitin-like proteins, which form a megacomplex with FtsH hexamers, was suggested in *E. coli* (Kihara et al., 1996; Saikawa et al., 2004). The interaction between prohibitin-like proteins and FtsH has been observed in cyanobacteria (Boehm et al., 2012). However, such prohibitin-like proteins have not been reported in chloroplasts, suggesting that thylakoid FtsH may have acquired other regulatory mechanisms along the endosymbiotic process.

Recent studies suggest that an increased turnover rate of FtsH during light irradiation is important for FtsH-mediated protein homeostasis in chloroplasts (Zaltsman et al., 2005a; Li et al., 2017; Wang et al., 2017; Kato et al., 2018). In the chloroplast of seed plants, thylakoid FtsH complex is rather unstable, and FtsH seems to exist in smaller complexes such as dimers (Yoshioka et al., 2010; Kato et al., 2018). The higher turnover rate of FtsH with flexible oligomerization seems to be required for the quality control of itself to access the damaged PSII complex, where ROS might generate at a high rate. On the other hand, disulfide bonds of FtsH controlled by the redox state are involved in the regulation of FtsH oligomerization and its proteolytic activity in *Chlamydomonas reinhardtii* (Wang et al., 2017).

Protein phosphorylation is one of the most important post-translational modifications (PTMs) in thylakoid membranes (Grieco et al., 2016). For example, phosphorylation of PSII core proteins and light-harvesting antenna proteins (LHCII), which are the best-characterized phosphorylated proteins in the thylakoid membrane, undergoes light-dependent regulation. These proteins are dephosphorylated in dark condition and rapidly phosphorylated under low to moderate light irradiation. Under a high light condition, the phosphorylation of PSII core proteins shows a light-dependent increase, whereas LHCII phosphorylation drastically decreases (Rintamäki et al., 1997; Tikkanen et al., 2006). The phosphorylation of these photosynthetic proteins contributes to the fine-tuning of the photosynthetic apparatus to achieve rapid photosynthetic acclimation. The phosphorylation of LHCII, which needs for state transition, is regulated by the STN7 kinase and PPH1 phosphatase pair (Bellafiore et al., 2005; Bonardi et al., 2005; Pribil et al., 2010; Shapiguzov et al., 2010). On the other hand, the reversible phosphorylation of PSII core proteins is regulated by the STN8 kinase–PBCP phosphatase system, being responsible for both positive and negative modulation of D1 degradation (Bonardi et al., 2005; Tikkanen et al., 2008; Samol et al., 2012; Kato and Sakamoto, 2014; Puthiyaveetil et al., 2014a). Recent studies reported the potential phosphorylation of FtsH proteases in the chloroplasts (Stael et al., 2012 and PhosPhAt 4.0 database). In contrast to extensive studies related to the involvement of PSII core proteins in PSII repair, the role of FtsH phosphorylation in that process, however, has been scarcely addressed and remains unclear.

In this study, we attempted to assess the effect of FtsH phosphorylation on this metalloprotease function. Aiming at that goal, we investigated the potential phosphorylation in FtsH by phosphate-affinity gel electrophoresis using a Phos-Tag molecule (Phos-tag SDS-PAGE) followed by immunoblot analysis. Both types of FtsH subunits were separated into phosphorylated and non-phosphorylated forms. We found that phosphorylation of thylakoid FtsH was neither dependent on light exposure nor the presence of the major thylakoid protein kinases, STN7 and STN8. Instead, we found that the phosphorylation status of FtsH varied with FtsH oligomerization degree. Furthermore, the leaf-variegated phenotype of *var2* was not rescued by expressing a mutated FtsH2 protein, harboring an amino acid substitution in the predicted phosphorylation site. These results imply that FtsH phosphorylation does not regulate FtsH function but FtsH complex formation or its stability.

## Materials and Methods

### Plant Materials and Growth Conditions

*Arabidopsis* (*Arabidopsis thaliana*) Columbia ecotype was used as wild type. The mutant lines used in this study, *stn7-1*, *stn8-1*, and *var2-1*, were described previously (Bellafiore et al., 2005; Bonardi et al., 2005; Vainonen et al., 2005; Kato and Sakamoto, 2014). Plants were germinated and grown on 0.7% (w/v) agar plates containing Murashige and Skoog medium supplemented with Gamborg’s vitamins (Sigma-Aldrich Corp.) and 1.5% (w/v) sucrose under 12-h light (approximately 100 µmol photons m^–2^ s^–1^), at a constant temperature of 22°C. The potential phosphorylation sites in FtsH2 were searched in the *Arabidopsis* Protein Phosphorylation Site Database (PhosPhAt 4.0; http://phosphat.mpimp-golm.mpg.de/).

For creating transgenic plants expressing mutated FtsHs under *var2-1* background, vector constructions were prepared as follows, using the primers shown in **Supplementary Table S1**. Amino acid substitutions in FtsH2 at the putative phosphorylation sites, S292A, T337A, S380A, and S393A, were introduced into the corresponding *VAR2* cDNA by PCR-based site-directed mutagenesis, using the appropriate set of primers (e.g. VAR2 Infusion-Fw/VAR2 S212A-Rv and VAR2 S212A-Fw/VAR2 Infusion-Rv, for S212A). The resulting PCR fragments were cloned into the BamHI-SacI site of a binary vector pBI121 using the In-fusion HD cloning kit (Takara Bio, USA). Final constructs were each subjected to sequencing, to confirm the accurate mutations inserted in the corresponding region. The constructs were transformed into *Agrobacterium tumefaciens* strain LBA4404 cells, and the positive LBA4404 strains were used to transform *var2-1* plants. Transgenic plants were selected by kanamycin resistance, and confirmed by PCR analysis (**Supplementary Figure S5**). At least three independent lines were obtained.

### Sample Preparation and Phos-Tag SDS-PAGE

Isolated thylakoid membranes were used for Phos-tag SDS-PAGE. Harvested 4-week-old seedlings were ground in a blender with ice-cold homogenization buffer (0.35 M sucrose, 50 mM HEPES, pH 7.0, 5 mM MgCl_2_, 10 mM NaCl, 10 mM NaF) containing EDTA-free complete protease inhibitor (Roche). Homogenates filtered through miracloth (Merck Millipore) were centrifuged at 2,380 × *g* for 10 min. The pellet was resuspended in the same buffer and centrifuged at 300 × *g* for 1 min. The supernatant was again centrifuged at 2,380 × *g* for 10 min. The pellet was resuspended in 1 x SDS-PAGE sample buffer and used for Phos-tag SDS-PAGE analyses. For phosphatase treatment, the thylakoid membranes were resuspended in 1 x Takara buffer for CIAP (supplied with CIAP) with cOmplete™ EDTA-free protease inhibitor (Merck) to a final chlorophyll concentration of 1 µg/µl. Resuspended thylakoid membranes were incubated with or without CIAP phosphatase for 2 h at 37°C. Phosphorylation of thylakoid membrane proteins was analyzed by Phos-tag SDS-PAGE. Phos-Tag™ was purchased from FUJIFILM Wako Pure Chemical Corporation and used according to the manufacturer’s protocol. In brief, gels for Phos-tag SDS-PAGE consisted of a separating gel [10% (w/v) acrylamide, 350 mM Bis-Tris, pH 6.8, 25 μM Phos-tag acrylamide, 100 μM ZnCl_2_, 0.1% (v/v) N,N,N’,N’-tetramethylethylenediamine (TEMED), and 0.05% (w/v) ammonium persulfate (APS)] and a stacking gel [4.5% (w/v) acrylamide, 350 mM Bis–Tris, pH 6.8, 0.1% (v/v) TEMED, and 0.05% (w/v) APS]. Electrophoresis was performed at a constant current of ≤20 mA/gel with the running buffer [100 mM Tris, 100 mM MOPS, and 0.1% (w/v) SDS], to which 5 mM of sodium bisulfite was added. For immunoblot analysis, gels were washed in wash buffer [25 mM Tris, 192 mM glycine, 0.1% (v/v) SDS, 10 mM EDTA] for 10 min three times to remove metal ions, followed by one wash in transfer buffer [25 mM Tris, 192 mM glycine, 0.1% (v/v) SDS, 20% methanol] for 10 min. Then, the proteins were electroblotted to the polyvinylidene difluoride (PVDF) membrane (ATTO Corporation).

### Two Dimensional (2D) Phos-Tag SDS PAGE

Purified thylakoid membranes were used for two-dimensional Phos-tag SDS-PAGE. Purified thylakoid membranes were resuspended in buffer [25 mM Bis–Tris, 20% (w/v) glycerol, pH 7.5] to a concentration of 2 mg/ml chlorophyll. To solubilize thylakoid membranes, an equal volume of n-dodecyl ß-D-maltoside was added to a final concentration of 1% (w/v). After centrifugation at 14,000 × *g* for 5 min, NativePAGE™ 5% G-250 Sample Additive (Thermo Fisher Scientific Inc.) was added to the supernatant according to the manufacturer’s instructions, and samples were loaded onto a NativePAGE™ 4–16% Bis–Tris Protein gel (Thermo Fisher Scientific Inc.) Electrophoresis was performed at 4°C overnight at 50 V. The gel lane was then excised from the gel and incubated in equilibration buffer [50 mM Tris–HCl, 6 M urea, 2% (w/v) SDS, 0.05% (w/v) BPB, 10 mM dithiothreitol] for 30 min at 37°C. Then proteins were separated using Phos-tag SDS-PAGE or conventional SDS-PAGE.

### Immunoblot Analysis

Prior to immunoreaction, transferred membranes were blocked with 1% (w/v) bovine serum albumin (BSA) in 50 mM sodium phosphate buffer, pH 7.5, containing 155 mM NaCl and 0.05% (v/v) Tween 20 (PBST buffer) for 1 h. The membranes were then incubated with anti-D1 (dilution 1:5,000), anti-VAR2 (dilution 1:5,000), anti-VAR1 (dilution 1:5,000), anti-CP43 (Agrisera; dilution, 1:5,000), anti-CP47 (Agrisera; dilution, 1:5,000), and anti-Lhcb1 (Agrisera; dilution, 1:5,000). After two washes with PBST buffer, the membranes were incubated with secondary antibodies Amersham ECL Rabbit IgG, HRP-linked F(ab)2 fragment from donkey (GE Healthcare; dilution, 1:10,000 in PBST). Luminata Forte Western HRP Substrate (EMD Millipore Corp.) was used to develop blots, and chemiluminescence was detected on ChemiDoc XRS+ System (Bio Rad Laboratories, Inc.).

### Fluorescence Measurements

Chlorophyll fluorescence was measured in mature leaves of 6-week-old plants using FluorCam800MF (Photon Systems Instruments). To induce photodamage, leaves were incubated for 4 h under high-light conditions (White LED light; 1,200 µmol photons m^–2^ s^–1^). Before measurement, leaves were dark-adapted for 10 min to oxidize plastoquinone. Initial fluorescence yield of PSII (F_o_) and maximal fluorescence yield of PSII (F_M_) were measured. Maximal PSII quantum yield (F_V_/F_M_) was calculated as (F_M_–F_o_)/F_M_.

## Results

### Detection of Phosphorylated FtsH in Thylakoid Membranes

**Figure 1:**
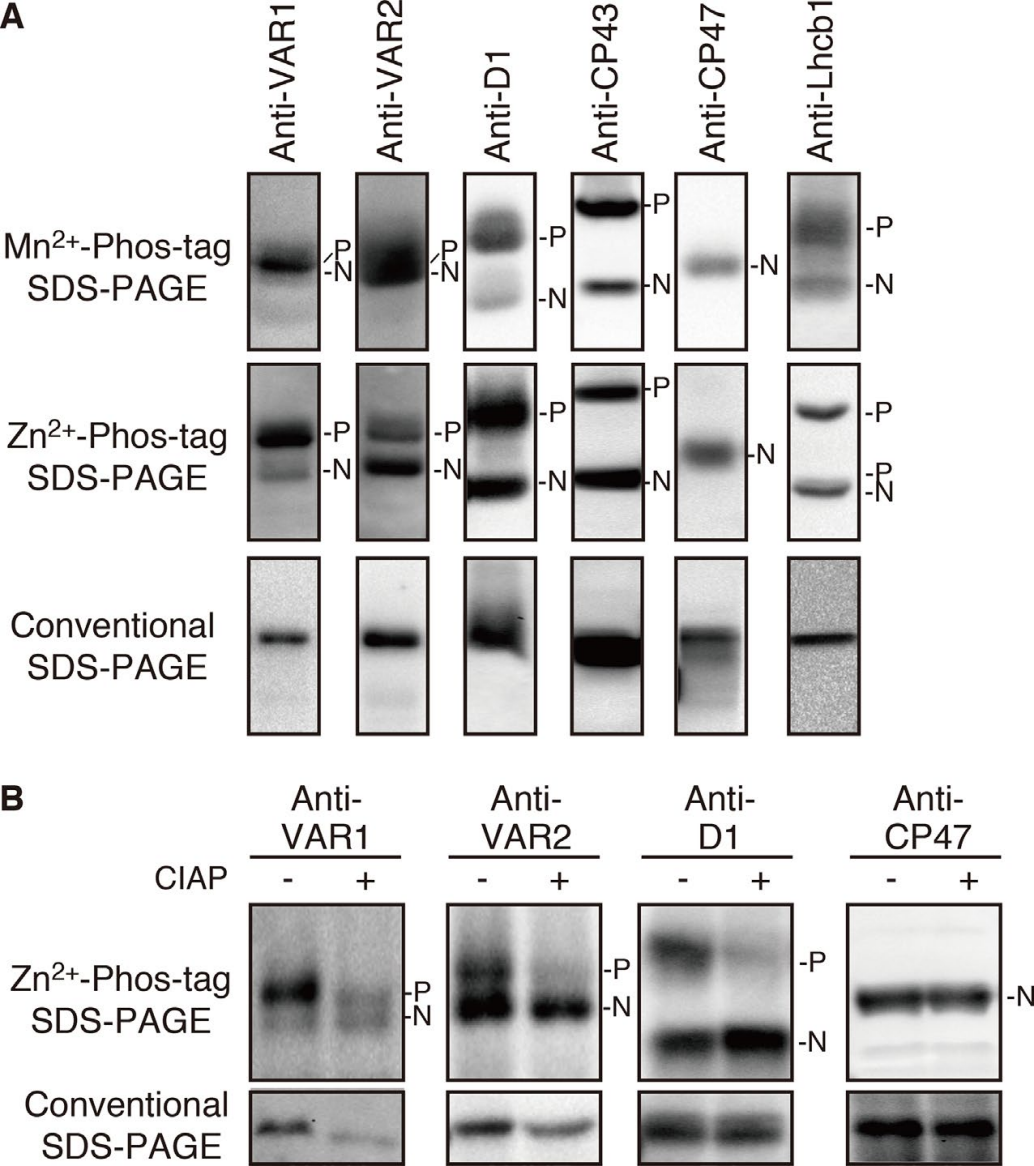
Mobility shift detection of phosphorylated and non-phosphorylated thylakoid proteins. **(A)** Thylakoid membrane proteins were isolated from 4-week-old seedlings and subjected to Mn^2+^–Phos-tag, Zn^2+^–Phos-tag, and conventional (Phos-tag-free) SDS–PAGE analyses. Immunoblot analyses were performed using anti-VAR1, anti-VAR2, anti-D1, anti-CP43, anti-CP47, and anti-Lhcb1 antibodies. **(B)** Purified thylakoid membranes were incubated with or without calf intestinal alkaline phosphatase (CIAP) and then subjected to Zn^2+^–Phos-tag SDS–PAGE and conventional SDS–PAGE analyses. Immunoblot analyses were performed using anti-VAR1, anti-VAR2, anti-D1, and anti-CP47 antibodies. The phosphorylated form (P) and non-phosphorylated form (N) are indicated. Proteins were loaded equally based on total chlorophyll content. The band density was measured with the ImageJ and showed in **Supplemental Figure S1**.

To evaluate potential phosphorylation in FtsH, we employed a phosphate-affinity gel electrophoresis system using a Phos-Tag molecule and subsequent immunoblot analysis. We first tested Phos-tag SDS-PAGE with Mn^2+^ as a chelating divalent cation using purified thylakoid membranes. The detected signals by immunoblotting using anti-VAR1 and VAR2 specific antibodies, which recognize type A and type B subunits, respectively, showed blurred signals of FtsH proteins, whereas phosphorylated PSII core proteins (D1 and CP43) and a light-harvesting antenna protein (Lhcb1) were properly resolved and separated from their unphosphorylated forms (**Figure 1A**). To improve the mobility shift of FtsH proteins, we next tested the Phos-tag SDS-PAGE procedure using Zn^2+^ instead of Mn^2+^, with a neutral pH buffer system (Kinoshita and Kinoshita-Kikuta, 2011). The result shown in **Figure 1A** indicated that the improved Phos-tag method successfully enabled us to detect both the phosphorylated and non-phosphorylated forms of FtsH proteins. A majority of the signals detected by anti-VAR1 antibodies (corresponding to type A FtsH) was observed in the up-shifted band, suggesting that type A subunits are extensively phosphorylated. Meanwhile, the phosphorylated form of type B FtsH detected by anti-VAR2 antibodies was less than half respect to total immunoblot signals (**Figure 1A**). These results are consistent with the different susceptibility of recombinant FtsH5 and FtsH2 found in calcium-dependent phosphorylation assays in a previous report (Stael et al., 2012). In contrast, a non-phosphorylated PSII core protein CP47 did not show any mobility shift in both electrophoresis conditions. The mobility shifts of FtsH proteins were substantially reduced after treatment with calf intestine alkaline phosphatase (CIAP), suggesting that the up-shifted band in the gel indeed resulted from phosphorylation (**Figure 1B**). Additionally, we found unexpectedly that FtsH levels were decreased in phosphatase-treated samples as compared to untreated samples. Since the levels of the non-phosphorylated form of FtsH were not significantly altered after phosphatase treatment, the decrease in FtsH total content seemed to reflect degradation of dephosphorylated-FtsH.

### Phosphorylation of FtsH Under Various Light Conditions and in the Kinase Mutants

**Figure 2:**
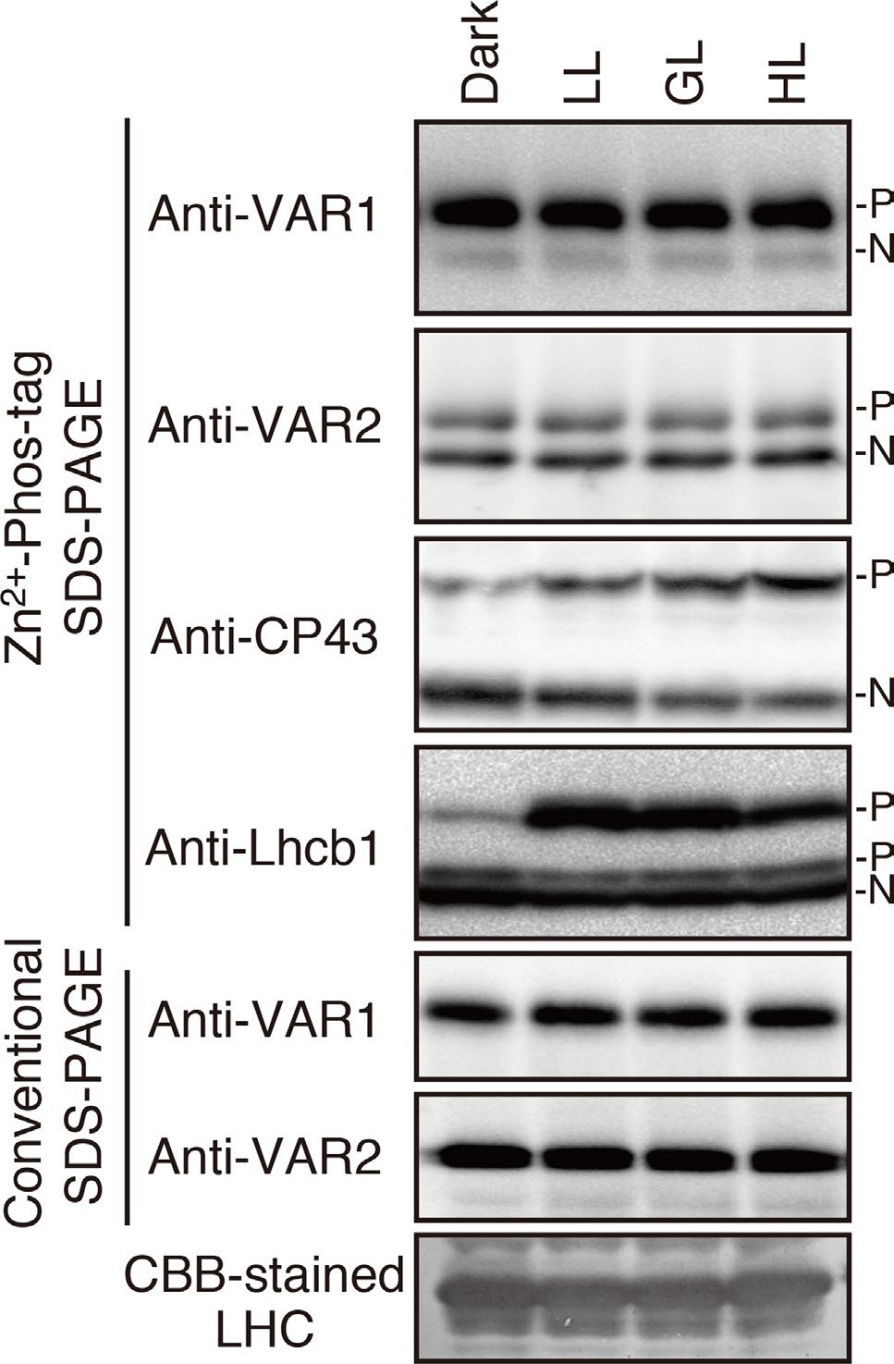
Phosphorylation of thylakoid proteins under various light conditions. Thylakoid membrane proteins were isolated from dark-adapted seedlings or seedlings exposed for 2 h to low (5 μmol photons m^–2^ s^–1^) (LL), growth (100 μmol photons m^–2^ s^–1^) (GL), or high (800 μmol photons m^–2^ s^–1^) (HL) light. Proteins were separated by Zn^2+^–Phos-tag SDS-PAGE and immunoblotted with anti-VAR1, anti-VAR2, anti-CP43, and anti-Lhcb1 antibodies. Conventional (Phos-tag-free) SDS–PAGE analysis was also carried out. The phosphorylated form (P) and non-phosphorylated form (N) are indicated. Proteins were loaded equally based on total chlorophyll content. (A Coomassie Brilliant Blue-stained gel of the same samples is shown at the bottom; LHC: light harvest complex). The band density was measured with the ImageJ and showed in **Supplemental Figure S2**.

Since FtsH is involved in the PSII repair resulting from light-dependent photo-damage (Lindahl et al., 2000; Bailey et al., 2002; Sakamoto et al., 2002; Sakamoto et al., 2003; Kato et al., 2009), we assessed whether the phosphorylated state of FtsH was affected by light conditions. Thylakoid proteins obtained from 4-week-old seedlings preincubated in the dark overnight and exposed to low- (5 µmol photons m^–2^ s^–1^), growth- (100 µmol photons m^–2^ s^–1^), or high-light (800 µmol photons m^–2^ s^–1^) for 2 h were subjected to Phos-tag SDS-PAGE and immunoblotting analysis. The results demonstrated that the phosphorylation level of PSII core protein CP43 increased in a light-dependent manner (**Figure 2**). On the other hand, Lhcb1 had few detectable phosphorylations in the dark incubation; the phosphorylation form increased upon low-light illumination, whereas diminished in the high-light irradiation. These results were consistent with those obtained in the previous studies (Rintamäki et al., 1997; Vener et al., 1998; Tikkanen et al., 2006). However, no obvious change in the phosphorylation state of FtsH proteins could be detected (**Figure 2**), suggesting that phosphorylation of FtsH is independent of the light-dependent regulation of photosynthesis-related proteins.

**Figure 3:**
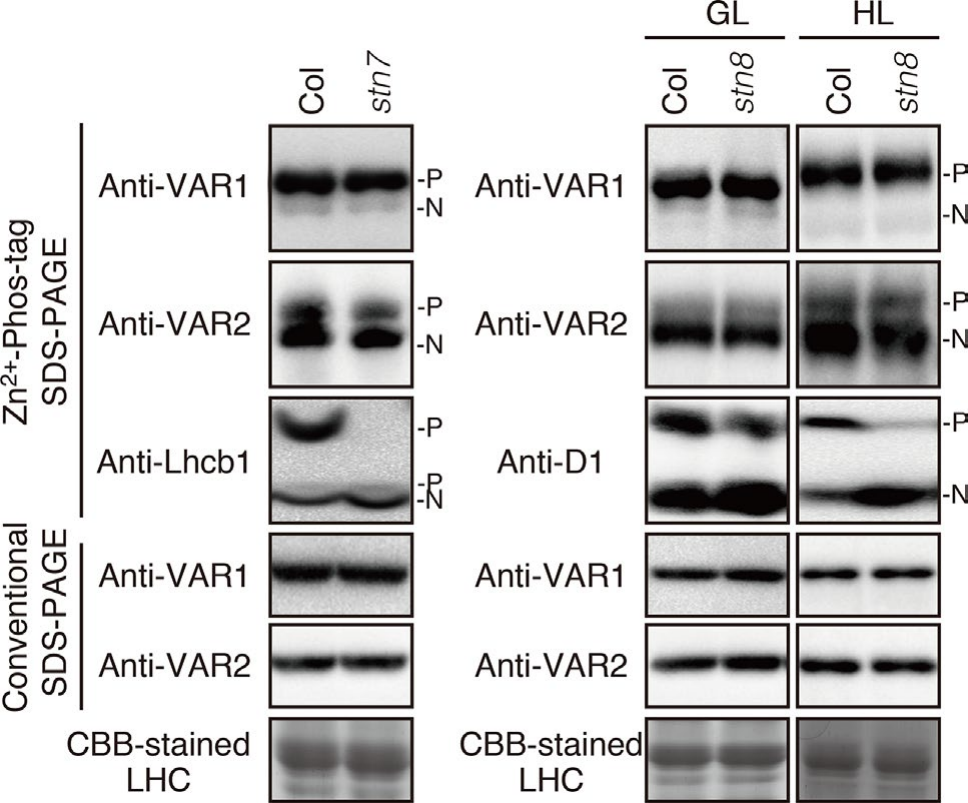
Phosphorylation of FtsH proteins in the kinase mutants. Thylakoid proteins were isolated from 4-week-old seedlings of wild-type (Col) and mutants lacking either STN7 (*stn7*) or STN8 (*stn8*). For additional analysis of protein phosphorylation in the *stn8* mutant, seedlings were exposed for 2 h to high-light (800 μmol photons m^–2^ s^–1^). The proteins were separated by Zn^2+^–Phos-tag SDS-PAGE and immunoblotted with anti-VAR1, anti-VAR2, anti-Lhcb1, and anti-D1 antibodies. Proteins were loaded equally based on total chlorophyll content. The phosphorylated form (P) and non-phosphorylated form (N) are indicated. The band density was measured with the ImageJ and showed in **Supplemental Figure S3**.

To clarify the relationship between the phosphorylation state of FtsH and light-dependent kinase activities in thylakoid membranes, we further evaluated the effect of two major thylakoid kinases, STN7 and STN8, on FtsH phosphorylation. STN7 is a thylakoid-associated kinase responsible for LHCII phosphorylation, which is required for state transition (Bellafiore et al., 2005; Bonardi et al., 2005). On the other hand, the phosphorylation of PSII core proteins is mainly regulated by STN8 kinase, but some degree of PSII core proteins phosphorylation is observed in the *stn8* knockout mutant due to an overlap in substrate specificity of STN7 and STN8 (Bonardi et al., 2005; Vainonen et al., 2005). In 4-week-old seedlings exposed to growth light (100 µmol photons m^–2^ s^–1^), the major phosphorylation band of Lhcb1 in *stn7*-mutant thylakoids was drastically decreased below the detection level (**Figure 3**), while minor phosphorylation band of Lhcb1 in a lower position was not affected in *stn7* mutant. A previous study reported that there is STN7 independent phosphorylation of a serine residue in Lhcb1; the serine residue corresponds to Ser-48 in Lhcb1.1 protein (Ingelsson and Vener, 2012). It seems that Phos-tag SDS-PAGE allows for detection of serine phosphorylation in Lhcb1. On the other hand, the phosphorylation level of FtsH proteins was comparable with that detected in control samples. Additionally, the band intensity of phosphorylated FtsH proteins obtained from *stn8*-mutant seedlings showed no difference compared with that of control seedlings under growth-light condition (100 µmol photons m^–2^ s^–1^). Phosphorylation of the PSII core protein D1 was still observed in this experimental condition, suggesting that the aforementioned phenomenon of overlapping activity of these two major thylakoid kinases effectively operated under this light intensity. However, under high-light condition (800 µmol photons m^–2^ s^–1^), the phosphorylation level of D1 proteins in the *stn8*-mutant was remarkably reduced (**Figure 3**), indicating that the phosphorylation of photosynthetic proteins was predominantly mediated by STN8 kinase in this irradiance situation. Nevertheless, the phosphorylation state of FtsH in *stn8*-mutant thylakoids showed no difference compared with that in control samples. These results suggested that STN8 is not involved in phosphorylation of FtsH.

### Phosphorylation State and FtsH Oligomerization

**Figure 4:**
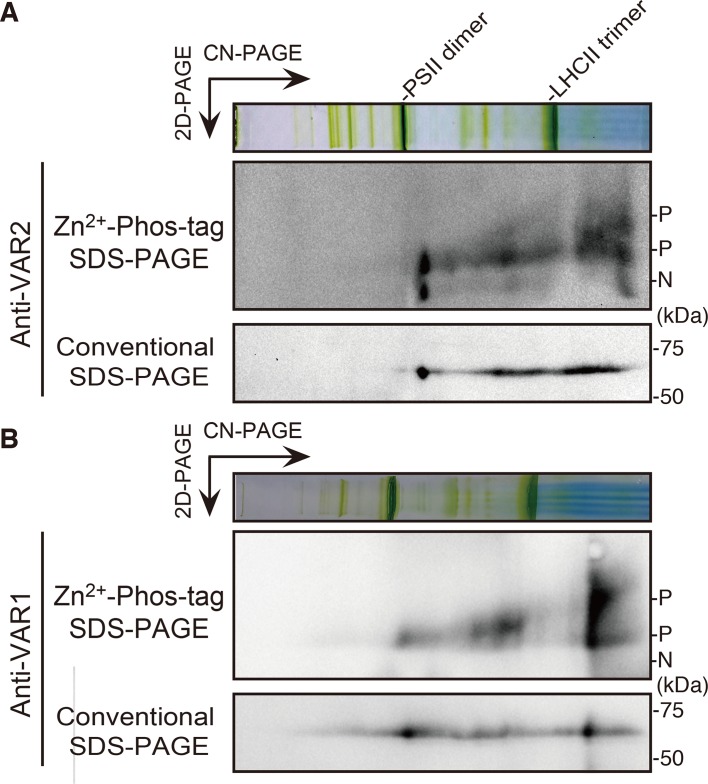
FtsH phosphorylation in different oligomeric complexes. Thylakoid membrane proteins were isolated from 4-week-old seedlings and solubilized with 1% n-dodecyl ß-D-maltoside. Solubilized protein complexes were subjected to clear-native (CN) PAGE at the first dimension. The gel lane was then subjected to Zn^2+^–Phos-tag and conventional SDS–PAGE analyses at the second dimension. Immunoblot analyses were performed using anti-VAR2 **(A)** and anti-VAR1 **(B)** antibodies. PSII dimer and LHCII trimer protein complexes positions are indicated at the CN-PAGE. The phosphorylated form (P) and non-phosphorylated form (N) are indicated. Representative results from three biological replicates (anti-VAR2) and two biological replicates (anti-VAR1) are shown.

Unlike bacterial FtsH megacomplexes, the FtsH hexamer complex in *Arabidopsis* chloroplasts was considered to be unstable, giving rise to smaller oligomers detected in a native PAGE (Li et al., 2017; Kato et al., 2018). To investigate the relation, if any, between phosphorylation and the oligomerization of FtsH, thylakoid membrane preparations solubilized in 1% n-dodecyl-β-D-maltoside were subjected to two-dimensional (2D) gel electrophoresis. Protein complexes were separated by clear-native (CN) PAGE in the first dimension and further separated by Zn^2+^-Phos-tag SDS-PAGE or conventional SDS-PAGE in the second dimension. The migration of FtsH proteins was resolved by immunoblotting using anti-VAR2 (for Type B) and anti-VAR1 (for Type A) antibodies. Conventional 2D-CN-SDS-PAGE showed that FtsH proteins migrated broadly from the higher molecular weight position, that was around PSII dimer, approximately 650 kDa, to the lower molecular weight size in a range of less than 140 kDa, estimated by the size of LHCII trimer complex (**Figures 4A, B**). Since the predicted molecular mass of FtsH monomer is about 70 kDa, the immunoblot signal at the smallest molecular size and that around LHCII trimer seemed to be the monomer and the dimer of FtsH, respectively. On the other hand, the phosphorylation pattern obtained after 2D electrophoresis using Phos-tag showed a noticeable change, particularly in the ratio of non-phosphorylated forms to phosphorylated forms in the Type B FtsH oligomers (**Figure 4A**). The immunoblot bands in the 2D CN PAGE showed that the phosphorylated form of FtsH migrated broadly, whereas high accumulation of the non-phosphorylated form of Type B FtsH was observed in the higher molecular weight and monomer positions. In addition, a portion of FtsH in the position corresponding to the dimeric and monomeric forms showed further retarded migration. Distribution of Type A FtsH assessed by anti-VAR1 antibodies showed the similar pattern to that of Type B FtsH, indicating that various oligomeric forms are detected in out 2D gel system (**Figure 4B**). In contrast to Type B, however, the non-phosphorylated forms strongly accumulated in the hexameric and monomeric regions were not noticeably observed. We reasoned that Type A FtsHs are extensively phosphorylated (**Figures 1** and **3**) and are not detectable in our gel system.

### Amino Acid Substitution of Potential Phosphorylated Residues in FtsH2

**Figure 5:**
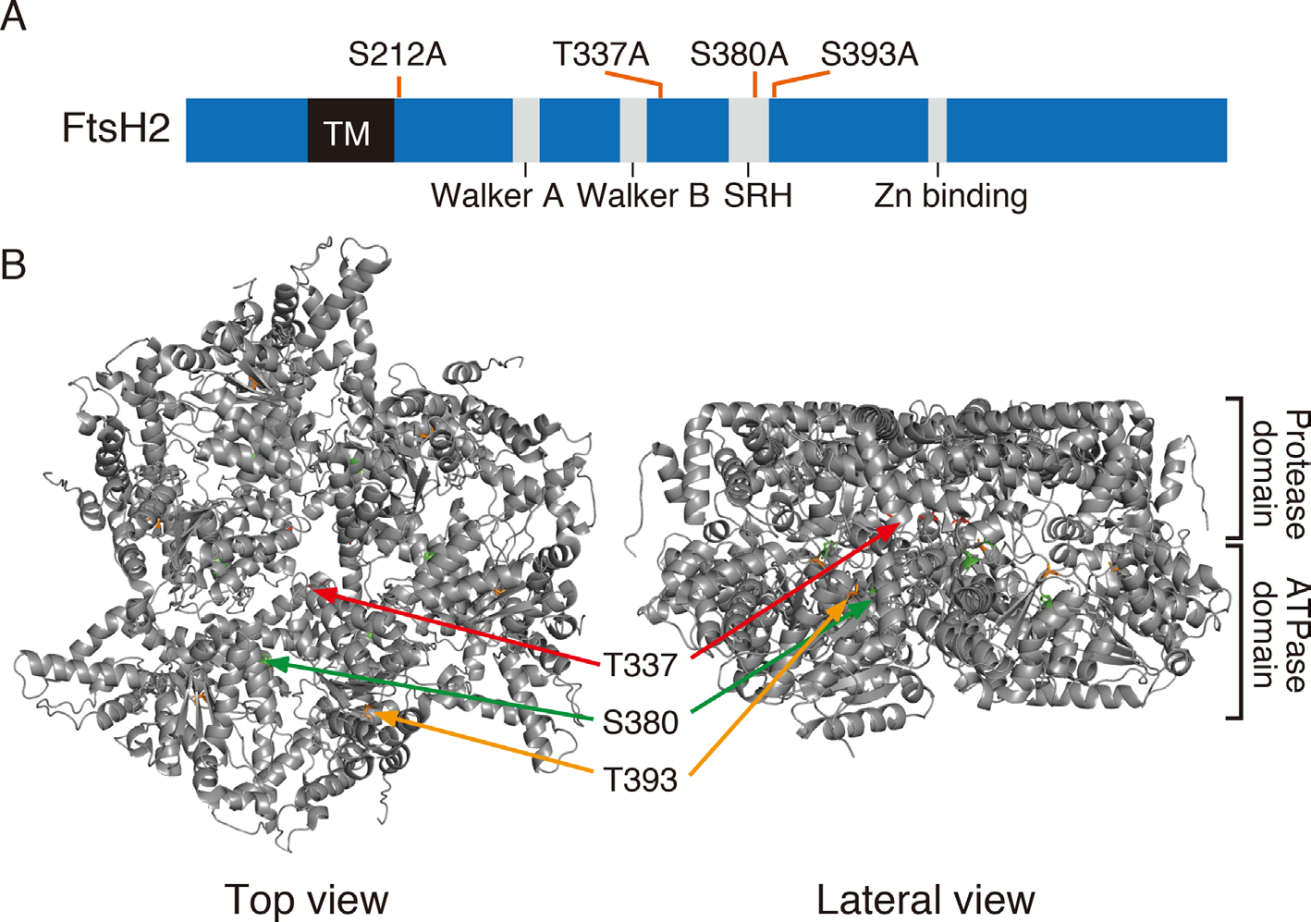
FtsH2 protein and possible phosphorylation sites. **(A)** Schematic representation of *Arabidopsis* FtsH2 protein as a horizontal bar, with its transmembrane domain (TM), their conserved motifs in the ATPase domain (Walker A, Walker B, and SRH), and the catalytic domain of Zn metalloprotease (Zn-binding site). Putative phosphorylated amino acids previously reported for FtsH2 (PhosPhAt Database) and assessed in this study are indicated above the bar. **(B)** Structure of FtsH hexamer in *Thermus thermophilus*, lacking the transmembrane domain. The positions corresponding to amino acids Thr-337, Ser-380, and Ser-393 of *Arabidopsis* FtsH are indicated (Thr-337 in red, Ser-380 in green, and Ser-393 in orange).

**Figure 6:**
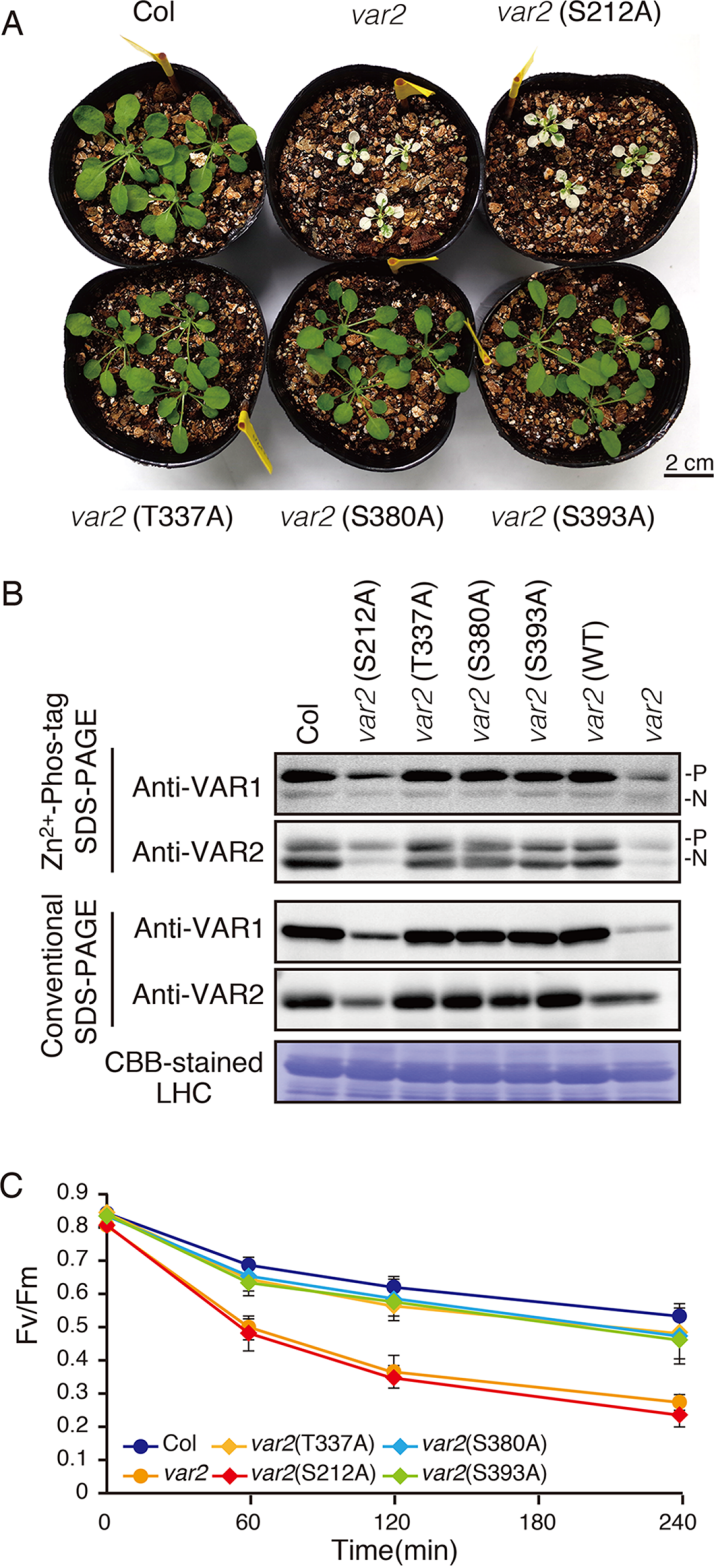
Complementation analyses of *var2* with mutated FtsH2 plants. **(A)** Photographs of 4-week-old Col, *var2-1*, *var2* (S212A), *var2* (T337A), *var2* (S380A), and *var2* (S393A) seedlings. Bars = 2 cm. **(B)** Accumulation and phosphorylation state of FtsH proteins. Thylakoid membrane proteins were isolated from 4-week-old seedlings and subjected to Zn^2+^–Phos-tag and conventional SDS–PAGE analyses. Immunoblot analyses were performed using anti-VAR1 and anti-VAR2 antibodies. Proteins were loaded equally based on total chlorophyll content. The phosphorylated form (P) and non-phosphorylated form (N) are indicated. A Coomassie Brilliant Blue-stained gel of the samples is shown at the bottom. **(C)** PSII photosensitivity during high light irradiation. Leaf discs obtained from mature leaves of Col, *var2-1*, *var2* (S212A), *var2* (T337A), *var2* (S380A), and *var2* (S393A) seedlings were exposed to high light (1,200 μmol photons m^–2^ s^–1^) and maximal fluorescence yields of PSII (F_M_) at 0, 60, 120, and 240 min after irradiation were determined. F_V_/F_M_ values were calculated as indicated in *Materials and Methods* (Data were obtained from six replicates; means ± SD are shown).

In the database of *Arabidopsis* large-scale phosphoproteomic studies (PhosPhAt 4.0) (Heazlewood et al., 2008; Durek et al., 2010), four Ser or Thr residues at position Ser-212, Thr-337, Ser-380, and Ser-393 were reported to be phosphorylated in the FtsH2 mature protein (**Table 1**). Amino acid alignment is shown in **Supplementary Figure S4**. To evaluate whether these potential phosphorylated residues in FtsH2 influence FtsH function, we performed site-directed mutagenesis to create an amino acid substitution. As shown in **Figure 5A**, we made *FtsH2* constructs (driven by CaMV *35S* promoter) in which the sequences corresponding to each Ser or Thr residue were replaced with Ala, and the mutanted FtsHs. The resulting constructs were introduced into a FtsH2-knockout mutant *var2-1*. Transgenic lines were designated as *var2* (S212A), *var2* (T337A), *var2* (S380A), and *var2* (S393A), respectively. Our previous work indicated that overexpression of FtsH2 restores leaf variegation successfully and accumulates FtsH levels comparable to the wild type (Zhang et al., 2010), suggesting post-translational control of FtsH heterocompex accumulation. As shown in **Figure 6A**, 4-week-old plants indicated that three of these transgenic lines, *var2* (T337A), *var2* (S380A), and *var2* (S393S), rescued leaf variegated phenotype in *var2-1*, demonstrating that the corresponding amino-acid substitutions are tolerated to restore the defective activity of FtsH. However, we found that leaf variegation persisted in *var2* (S212A), suggesting that Ser-212 is important to maintain its activity (**Figure 6A**).

**Table 1:** Putative phosphorylation sites of thylakoid FtsH proteins in *Arabidopsis thaliana* and *Chlamydomonas reinhardtii*.

Gene	Protein	Peptide	No. pSTY	Putative phospho-site(s)	References
AT5G42270	AtFtsH5(VAR1)	SKFQEVPETGVTFGDVAGADQAK	1	S237, T245, T248	Reiland et al., 2009; Reiland et al., 2011
		SKSKFQEVPETGVTFGDVAGADQAK	1	S235, S237, T245, T248	
		QVTVDRPDVAGR	1	T416	Roitinger et al., 2015
AT1G50250	AtFtsH1	MASNSLLR	1	S5	Engelsberger and Schulze, 2012
		QVTVDRPDVAGR	1	T428	Roitinger et al., 2015
AT2G30950	AtFtsH2(VAR2)	AASSACLVGNGLSVNTTTKQR	3	S5, S14, T17	Mithoe et al., 2012
		QTSFSSVIR	1	S33	Roitinger et al., 2015
		SGGGMGGPGGPGNPLQFGQSK	1	S212	
		GTGIGGGNDER	1	T337	
		QRGTGIGGGNDER	1	T337	
		ADILDSALLRPGR	1	S380	
		QVSVDVPDVK	1	S393	
AT1G06430	AtFtsH8	SSGGMGGPGGPGFPLQIGQSKAK	1	S205	
		GTGIGGGNDER	1	T330	Roitinger et al., 2015
		QRGTGIGGGNDER	1	T330	
		ADILDSALLRPGR	1	S373	
		QVSVDVPDVK	1	S386	Roitinger et al., 2015; Bhaskara et al., 2017
		IVAGMEGTVMTDGK	1	T467	Bhaskara et al., 2017
Cre17.g720050	CrFtsH2	QVSVDLPDQK	1	S382	Wang et al., 2014
		GGAELVAAATRMEL	1	T685	
		KGGAELVAAATRMEL	1	T685	

To examine if mutated FtsH2 proteins accumulated in the transgenic lines, thylakoid membrane proteins from 4-week-old seedlings were subjected to immunoblot analysis (**Figure 6B**). FtsHs accumulated to a similar extent in control, *var2* (T337A), *var2* (S380A), and *var2* (S393A) lines. By contrast, accumulation of FtsH in *var2* (S212A) was reduced considerably, whose levels appeared to be comparable to those in the original *var2* mutant. Since anti-VAR2 antibodies recognize type B FtsH, the remaining bands in *var2* samples were assumed to correspond to another type B protein, FtsH8. Therefore, it is likely that the lack of recovery from variegation in *var2* (S212A) was due to the impaired accumulation of FtsH2 in this line. On the other hand, Phos-tag SDS-PAGE demonstrated phosphorylation of FtsH in the three transgenic lines, *var2* (T337A), *var2* (S380A), and *var2* (S393S); their phosphorylation levels were comparable to that of control. To further evaluate whether these amino-acid substitutions affected FtsH activity in the thylakoid membrane, high-light sensitivity of PSII activity was measured using chlorophyll fluorescence analysis. As expected, an increased PSII photosensitivity was observed in *var2* (S212A), similar to that of *var2* mutant (**Figure 6C**). The F_V_/F_M_ values in *var2* (T337A), *var2* (S380A), and *var2* (S393S) lines slightly decreased with respect to that of the wild type during high-light irradiation, but these differences were not statistically significant. These results using transgenic plants were thus unable to confirm that phosphorylation of the predicted target residues plays a critical role in FtsH phosphorylation. Nevertheless, our data demonstrated that S212, localized at the stromal edge of transmembrane domain connecting the ATPase domain, is important for FtsH stability.

## Discussion

Protein phosphorylation of FtsH has been observed in large-scale comparative phosphoproteomics studies in *Arabidopsis* (**Table 1**). In addition, previous studies reported the phosphorylation of FtsH in isolated chloroplasts of pea and *Arabidopsis* (Stael et al., 2012), and also in *Chlamydomonas* (Wang et al., 2014; Szyszka-Mroz et al., 2015). Although several reports suggested the potential phosphorylation of FtsH in thylakoid membranes, its regulatory role remains to be elucidated. Over the past decades, phosphorylation of thylakoid proteins has been characterized as one of the most important regulatory mechanisms of photosynthesis. However, characterization of phosphorylated proteins, focusing on the particular protein target in thylakoid membrane by using immunoblot analysis, appears to have several limitations; for example, conventional immunoblot approaches using anti-phosphothreonine antibodies exclusively detect major phosphorylated proteins such as PSII core proteins and LHCII, which hampers detection of minor phosphorylation. To overcome this drawback, we attempted to evaluate FtsH phosphorylation based on a Phos-tag approach. In PAGE gels containing Phos-tag molecules (Phos-tag SDS-PAGE), Phos-tag interferes with the migration of phosphorylated proteins, thereby exhibiting them as a retarded band, in addition to its non-phosphorylated forms. We found that the use of Zn^2+^ as divalent cation enabled us to identify the phosphorylated form of FtsH in thylakoid membranes successfully (**Figure 1**).

Since photodamage of D1 in the PSII complex increases with higher light intensity, we may predict the existence of light-induced regulation that governs the proteolytic machinery for an efficient quality control of PSII activity. One of the lumenal serine-type Deg proteases, Deg1, which facilitates the effective D1 degradation by FtsH in photoinhibitry conditions, has a regulation mechanism dependent on thylakoid lumen acidification (Kley et al., 2011). However, our characterization under various light conditions showed that the phosphorylation state of FtsH in the thylakoid membranes did not undergo light-dependent regulation (**Figure 2**), suggesting that phosphorylation of FtsH is not important to increase proteolysis under high-light intensity. This idea was further supported by the finding that FtsH phosphorylation was not directly regulated by two major thylakoid kinases (**Figure 3**). FtsH phosphorylation is likely mediated by other ill-defined protein kinases localized in chloroplasts. Of note is that in several experiments, bands corresponding to phosphorylated-FtsH proteins from thylakoids of *stn7*-mutant seedlings slightly decreased than those observed in control seedlings; nevertheless, the reproducibility of this result was limited. Given that the loss of STN7 kinase affects both short and long-term photosynthetic acclimation (Pesaresi et al., 2009), we cannot exclude the possibility that indirect effects caused by the loss of STN7 influenced the phosphorylation state of FtsH.

It is unclear as to how FtsH function is regulated by light in the chloroplasts of *Arabidopsis*, apart from the light-induced transcription of FtsH8, which is likely to compensate for the rapid FtsH degradation caused by high-light (Sinvany-Villalobo et al., 2004; Zaltsman et al., 2005a; Wang et al., 2017; Kato et al., 2018). Previous studies demonstrated that FtsH selectively degrades D1 even in dark condition (Lindahl et al., 2000; Krynická et al., 2015), suggesting that light is not always a decisive factor for FtsH protease activity per se; rather, accessibility to their substrates may be crucial for FtsH-mediated protein degradation (Krynická et al., 2015). Together with our results, these observations raise the possibility that the structural change around D1 in the damaged PSII complex is probably more important for the increased proteolysis expected under high-light intensity than light-dependent induction of protease activity in FtsH (Lindahl et al., 2000; Krynická et al., 2015). This possibility well fits with the observation that light-induced unstacking of the grana expands the grana margin region in the thylakoid membrane; the expansion of grana margin seems to increase the accessibility of photodamaged D1 to FtsH (Puthiyaveetil et al., 2014b; Yoshioka-Nishimura et al., 2014). The alternative possibility may be the redox regulation of FtsH mediated by the formation of Cys disulfide bonding, as suggested in *Chlamydomonas* under high-light irradiation (Wang et al., 2017). Further investigation is necessary to study post-translational regulation of FtsH in *Arabidopsis* chloroplasts.

Amino acid substitutions carried out in FtsH2, based on information provided at the *Arabidopsis* Protein Phosphorylation Site Database (PhosPhAt 4.0), showed that Ser-212 is important for FtsH accumulation, whereas these residues did not critically affect the phosphorylation state of FtsH assessed by Phos-tag analysis (**Figure 6**). The N-terminal Ser-212 residue was found to be conserved between type A and type B subunits among photosynthetic organisms, whereas the Ser-380 in the second region of homology (SRH) motifs of ATPase domain is conserved between type A and type B subunits, but it is not found in FtsH proteins of other photosynthetic organisms (**Supplementary Figure S4**). Besides, Thr-337 and Ser-393 in the ATPase domain are conserved in the type B subunit of FtsH protein between *Arabidopsis* and *Chlamydomonas*, in contrast to type A subunit. The structural model of FtsH hexamer lacks the surrounding transmembrane domain including the position corresponding to Ser-212 (**Figure 5B**). Given the sequence homology among FtsH proteases, Ser-212 would locate between the transmembrane domain and the first alpha-helix in the ATPase domain. This connecting area would be sandwiched between thylakoid membranes and the stromal region of FtsH and seems to be important for substrate translocation and recognition. The phosphorylation of this region was also observed in FtsH5, although it remained unclear which amino acid residue was phosphorylated (**Table 1**). The finding of decreased accumulation of thylakoid FtsH after phosphatase treatment (**Figure 1B**) suggests the possible effect of phosphorylation on FtsH stability in the thylakoid membranes. However, the possibility that in the transgenic line *var2* (S212A), designed for Ser-212 amino acid substitution, this condition itself could have influenced the translation process or the stability of FtsH protein, cannot be ruled out.

In thylakoid membranes, the FtsH functional complex is likely a temporary complex formed when the proteolysis is executed in the grana margin region (Yoshioka et al., 2010; Kato et al., 2018). Thus, FtsH in thylakoid membranes probably does not form a megacomplex with other proteins but is mainly present as smaller complexes. This flexible oligomerization capability of FtsH in the chloroplasts might contribute to the turnover of FtsH itself to maintain its activity under high-light condition (Wang et al., 2017; Kato et al., 2018). Interestingly, our two-dimensional PAGE approach showed different phosphorylation states among FtsH oligomers (**Figure 4**): greater phosphorylation degree of FtsHseems to be found in smaller oligomers, suggesting that FtsH phosphorylation was somehow related to the stability of its monomeric and dimeric forms in the thylakoid membranes. However, the possibility that the phosphorylation state of FtsH affects the solubilization process of the FtsH complexes could not be excluded. On the other hand, it is still unclear how calcium-dependent phosphorylation of FtsH, reported in a previous study (Stael et al., 2012), influences FtsH complex formation and function.

## Data Availability

All datasets generated for this study are included in the manuscript/**Supplementary Files**.

## Author Contributions

YK and WS conceived the study and wrote the manuscript. YK performed the experiments. All authors reviewed the results and approved the final version of the manuscript.

## Funding

This work was supported by KAKENHI grants from Japan Society for the Promotion of Science (16H06554 and 17H03699 to WS; 18K06290 to YK) and from the Oohara Foundation (to WS and YK).

## Conflict of Interest Statement

The authors declare that the research was conducted in the absence of any commercial or financial relationships that could be construed as a potential conflict of interest.
