# Supplementary material for: Phosphorylation of the Chloroplastic Metalloprotease FtsH in Arabidopsis Characterized by Phos-Tag SDS-PAGE
Source: Front Plant Sci. 2019 Sep 10;10:1080. doi: 10.3389/fpls.2019.01080 (PMC6747001; doi:10.3389/fpls.2019.01080)
Supplement: Supplementary file 1 [file DataSheet_1.pdf]

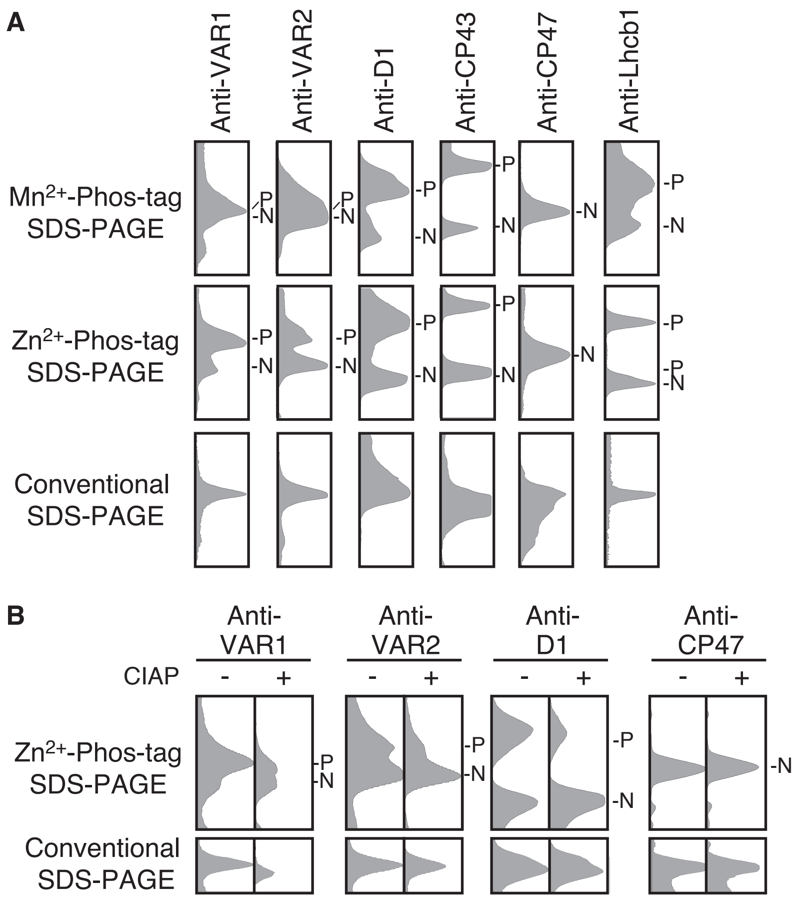

Suppramentary FIGURE 1. The band density of Figure 1. (A). Thylakoid membrane proteins were isolated from 4-week-old seedlings and subjected to Mn^{2+}–Phos-tag, Zn^{2+}–Phos-tag, and conventional (Phos-tag-free) SDS–PAGE analyses. (B) Purified thylakoid membranes were incubated with or without calf intestinal alkaline phosphatase (CIAP) and then subjected to Zn^{2+}–Phos-tag SDS–PAGE and conventional SDS–PAGE analyses. The band density was measured with the ImageJ software.

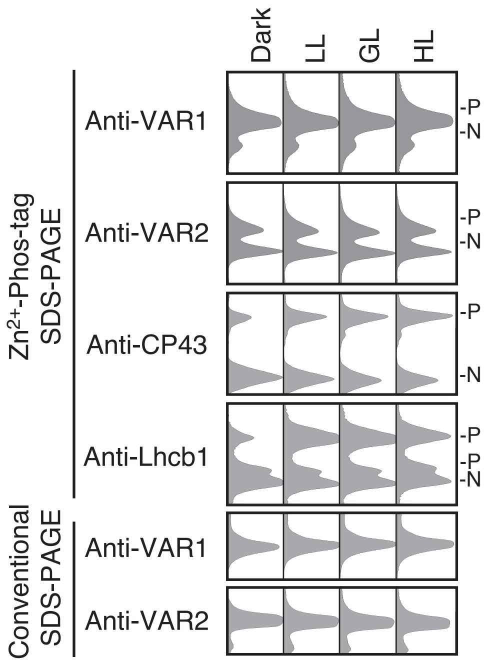

Suppementary FIGURE 2. The band density of Figure 2. Thylakoid membrane proteins were isolated from dark-adapted seedlings or seedlings exposed for 2 h to low (5 µmol photons m^{-2} s^{-1}) (LL), growth (100 µmol photons m^{-2} s^{-1}) (GL), or high (800 µmol photons m^{-2} s^{-1}) (HL) light. Proteins were separated by Zn^{2+}–Phos-tag SDS-PAGE and immunoblotted with anti-VAR1, anti-VAR2, anti-CP43, and anti-Lhcb1 antibodies. Conventional (Phos-tag-free) SDS–PAGE analysis was also carried out. The phosphorylated form (P) and non-phosphorylated form (N) are indicated. Proteins were loaded equally based on total chlorophyll content. The band density was measured with the ImageJ software.

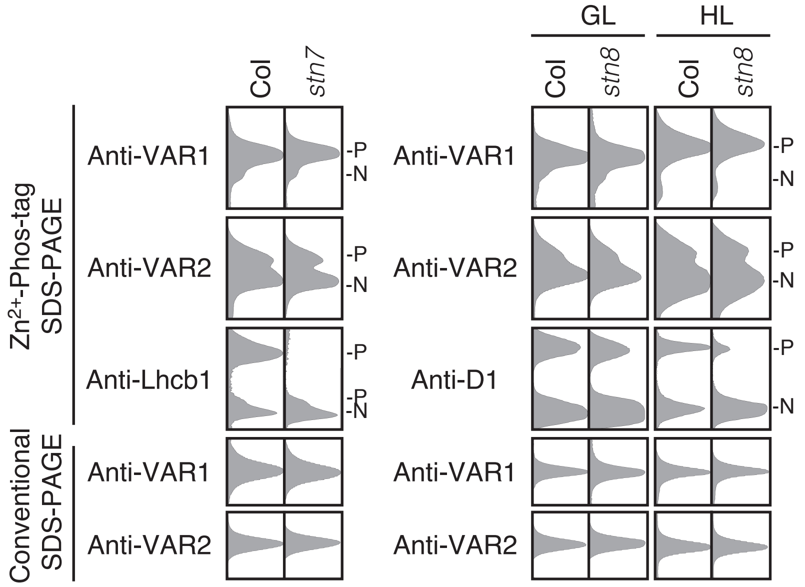

Suppremementary FIGURE 3. The band density of Figure 3. Thylakoid proteins were isolated from 4-week-old seedlings of wild-type (Col) and mutants lacking either STN7 (*stn7*) or STN8 (*stn8*). For additional analysis of protein phosphorylation in the *stn8* mutant, seedlings were exposed for 2 h to high-light (800 µmol photons m^{-2} s^{-1}). The proteins were separated by Zn^{2+}–Phos-tag SDS-PAGE and immunoblotted with anti-VAR1, anti-VAR2, anti-Lhcb1, and anti-D1 antibodies. Proteins were loaded equally based on total chlorophyll content. The phosphorylated form (P) and non-phosphorylated form (N) are indicated. The band density was measured with the ImageJ software.

```
AtFtsH1         1 -MASNSLLRSSSNFFLGSHIIISSPTPKTTRKPSFPFSFVSRAKYQITRSSQDENSPNGKPNSPFSSQVALAAILLSSIS  79
AtFtsH5 (VAR1)  1 MATTSSNPLLLSSNFLGSQIIISAPTPKTTTK-SLPFSVISRKRYQI--------SQSEKLMKSLPSQAALAALLFSSSS  71
CrFtsH1         1 ----MSAFTARPEALASKAGRRTFARNTEALRSQLLGLPKRSSRVTAKYSPKQLSENLASELVAKLPKAALASLLSLSTA  76
SynFtsH3        1 --------------------------------------------------------------------------------   1
AtFtsH2 (VAR2)  1 ------------------------------------------MAASSACLVGNGLSVNTTTKQRLSKHFSGRQTSFSSVIR  39
AtFtsH8         1 ------------------------------------------MAASSACLLGNGLSVYTT-KQRFQK---------LGLDR  29
CrFtsH2         1 -----------------------------------------------------MQMLQQRVVMRQARPAALKPVLPGALRT  28
SynFtsH1        1 ------------------------------------------------------MSHRPRSDRHSFSSPSRFWHRLGMGLL  27
SynFtsH2        1 --------------------------------------------------------------------------------   1

                       TPP                                      TPP
AtFtsH1        80 SSPLALAVVDEPASPSVVIESQAVKPSTPSPLFIQNEILKAPSPK--SSDLPEGSQWRYSEFLNAVKKGKVERVRFSKDG  157
AtFtsH5 (VAR1) 72 PQALAVNEPVQPPAPTITAEAQ----SPNLSTFGQNVLMTAPNPQAQSSDLPDGTQWRYSEFLNAVKKGKVERVKFSKDG  147
CrFtsH1        77 GVATAADYFAPPTDANTTTTTATTQAPASMN-FASSAPLAAPEVR-SEYTLPEGNQWRYSEFVNAVEAGKVERVRFSKDG  154
SynFtsH3        1 ------------------MSKNNKKWRNAGLYALLLIVVLALASAFFDRPTQTRETLSYSDFVNRVEANQIERVNLSADR  62
AtFtsH2 (VAR2) 40 TSKVNVVKASLDGKKKQEGRRDFLKILLGNA--GVGLVASGKANADEQG--VSSSRMSYSRFLEYLDKDRVNKVDLYENG  115
AtFtsH8        30 TSKVTVVKASLDEKK-HEGRRGFFKLLLGNAAAGVGLLASGNANADEQGQGVSSSRMSYSRFLEYLDKGRVEKVDLYENG  108
CrFtsH2        29 RAVVVRAQQEQQTEVVASGKRDLIRNAIAAA---VAVMPVMAAKAEDAA-GVASSRMSYSRFLEYLEMGRVKKVDLYENG  104
SynFtsH1       28 VAGTLALPVSTLAQEGGEGAQPKASPSPIQSPNSSNGEATPRSFFNSGSPRSAEPKMNYGQLIDAIKANQVAKVEVDTNR  107
SynFtsH2        1 --------------MKFSWRTALLWSLPLLVVGFFFWQGSFGGADANLGSNTANTRMTYGRFLEYVDAGRITSVDLYENG  66
                                                                                 . *. ..  .  . .. *  .  ..

                                                                                     TM
AtFtsH1       158 SVVQLTAVDNR------RASVI-VPNDPDLIDILAMNGVDISVSEG-ESSGNDLFTVIGNLIFPLLAFGGLFLLFRRAQ-  228
AtFtsH5 (VAR1)148 SVLQLTAVDNR------RATVI-VPNDPDLIDILAMNGVDISVSEG-EG-GNGLFDFIGNLLFPLLAFGGLFYLFRGGQ-  217
CrFtsH1       155 SQLQLTAVDGR------RATVV-LPNDPDLVDILAKNGVDISVSEG-DQQGNYV-ALLGNILFPLIAFGGLFFLFRRSQN  225
SynFtsH3       63 TQAQVPNPSGG------PPYLVNLPNDPDLINILTQHNVDIAVQPQ-SDEG-FWFRIASTLFLPILLLVGIFFLFRRAQ-  133
AtFtsH2 (VAR2)116 TIAIVEAVSPELGNRVERVRVQLPGLSQELLQKLRAKNIDFAAHNAQEDQGSVLFNLIGNLAFPALLIGGLFLLSRRS--  193
AtFtsH8       109 TIAIVEAVSPELGNRIQRVRVQLPGLSQELLQKLRAKNIDFAAHNAQEDQGSPILNLIGNLAFPVILIGGLFLLSRRS--  186
CrFtsH2       105 TIAIVEAVSPELGNRVQRVRVQLPGTSPELLGKFREKKIDFAAHANTEDGGAVFLNLLGNLAFPLLLVAGLFLLSRQS--  182
SynFtsH1      108 RQAIVTLKDAPPGSKPQ--TVQLLDNNPELLNLLRSRSETIDLDINRTPDNSALYGLLTNLLVVAILIGLVVMVVRRS--  183
SynFtsH2       67 RTAIVQVSDPEV-DRTLRSRVDLPTNAPELIARLRDSNIRLDSHPVRNN--GMVWGFVGNLIFPVLLIASLFFLFRRS--  141
                    ... ...        .  .  .  .  . ..*   ..    ..      .  .     . ... .. .. .... ..*..

                                                                                       Walker A
AtFtsH1       229 GGPGGGP-GGLGGPMDFGRSKSKFQEVPETGVSFADVAGADQAKLELQEVVDFLKNPDKYTALGAKIPKGCLLVGPPGTG  307
AtFtsH5 (VAR1)218 GGAGG-P-GGLGGPMDFGRSKSKFQEVPETGVTFGDVAGADQAKLELQEVVDFLKNPDKYTALGAKIPKGCLLVGPPGTG  295
CrFtsH1       226 GGGGAGPMGGMGGAMDFGRSKSKFQEVPETGVVFDDVAGCDGAKLELQEVVDFLKNPDKYTALGAKIPKGCLLVGPPGTG  305
SynFtsH3      134 ----SGP--G-SQAMNFGKSKARVQMEPQTQVTFGDVAGIEQAKLELTEVVDFLKNADRFTELGAKIPKGVLLVGPPGTG  206
AtFtsH2 (VAR2)194 GGGMGGP-GGPGNPLQFGQSKAKFQMEPNTGVTFDDVAGVDEAKQDFMEVVEFLKKPERFTAVGAKIPKGVLLIGPPGTG  272
AtFtsH8       187 SGGMGGP-GGPGFPLQIGQSKAKFQMEPNTGVTFDDVAGVDEAKQDFMEVVEFLKKPERFTAVGARIPKGVLLVGPPGTG  265
CrFtsH2       183 QGGMGGP-GNPNNPLNFGKSRARFQMEANTGVTFNDVAGVDEAKQDFMEIVEFLKRPERFTAVGARIPKGCLLVGPPGTG  261
SynFtsH1      184 ------A-NASGQAMSFGKSKARFQMEAKTGVGFDDVAGIDEAKEELQEVVTFLKQPEKFTAIGAKIPRGVLLIGPPGTG  256
SynFtsH2      142 -SNM--P-GGPGQAMNFGKSKARFQMDAKTGVMFDDVAGIDEAKEELQEVVTFLKQPERFTAVGAKIPKGVLLVGPPGTG  217
                   .  ... .. .  .. .* *....*...  *.*.*.****  ..**  ...*.* ***  ....*.  **.**.*.**.******

                                                                Walker B
AtFtsH1       308 KTLLARAVAGEAGVPFFSCAASEFVELFVGVGASRVRDLFEKAKSKAPCIVFIDEIDAVGRQRGAGMGGGNDEREQTINQ  387
AtFtsH5 (VAR1)296 KTLLARAVAGEAGVPFFSCAASEFVELFVGVGASRVRDLFEKAKSKAPCIVFIDEIDAVGRQRGAGMGGGNDEREQTINQ  375
CrFtsH1       306 KTLLAKAVAGEAGTPFFSCAASEFVEVFVGVGASRVRDLFEKAKSKAPCIIFIDEIDAVGRQRGAGMGGGNDEREQTINQ  385
SynFtsH3      207 KTLLAKAVAGEAGVPFFSISGSEFVEMFVGVGASRVRDLFEQAKANAPCIVFIDEIDAVGRQRGAGLGGGNDEREQTLNQ  286
AtFtsH2 (VAR2)273 KTLLAKAIAGEAGVPFFSISGSEFVEMFVGVGASRVRDLFKKAKENAPCIVFVDEIDAVGRQRGTGIGGGNDEREQTLNQ  352
AtFtsH8       266 KTLLAKAIAGEAGVPFFSISGSEFVEMFVGVGASRVRDLFKKAKENAPCIVFVDEIDAVGRQRGTGIGGGNDEREQTLNQ  345
CrFtsH2       262 KTLLAKAIAGEAGVPFFSVSGSEFVEMFVGVGASRVRDLFKKAKENAPCLVFVDEIDAVGRSRGTGIGGTNDEREQTLNQ  341
SynFtsH1      257 KTLLAKAIAGEAGVPFFSISGSEFVEMFVGVGASRVRDLFKKAKENAPCLVFIDEIDAVGRQRGVGYGGGNDEREQTLNQ  336
SynFtsH2      218 KTLLAKAIAGEAGVPFFSISGSEFVEMFVGVGASRVRDLFKKAKENAPCLIFIDEIDAVGRQRGAGIGGGNDEREQTLNQ  297
                  *****.*.*****.****...*****.************..**..***..*.********.**.* **.*******.**

                                  SRH
AtFtsH1       388 LLTEMDGFSGNSGVIVLAATNRPDVLDSALLRPGRFDRQVTVDRPDVAGRVKILQVHSRGKALGKDVDFDKVARRTPGFT  467
AtFtsH5 (VAR1)376 LLTEMDGFSGNSGVIVLAATNRPDVLDSALLRPGRFDRQVTVDRPDVAGRVQILKVHSRGKAIGKDVDYEKVARRTPGFT  455
CrFtsH1       386 LLTEMDGFEGNTGVIVLAATNRPDVLDQALLRPGRFDRQVTVDRPDVQGRVSILKVHSRGKALGKDVDLEKIARRTPGFT  465
SynFtsH3      287 LLTEMDGFEGNTGIIIVAATNRPDVLDSALMRPGRFDRQVVVDRPDYAGRREILNVHARGKTLSQDVDLDKIARRTPGFT  366
AtFtsH2 (VAR2)353 LLTEMDGFEGNTGVIVVAATNRADILDSALLRPGRFDRQVSVDVPDVKGRTDILKVHAGNKKFDNDVSLEIIAMRTPGFS  432
AtFtsH8       346 LLTEMDGFEGNTGVIVVAATNRADILDSALLRPGRFDRQVSVDVPDVKGRTDILKVHSGNKKFESGVSLEVIAMRTPGFS  425
CrFtsH2       342 MLTEMDGFEGNTGIIVIAATNRADILDPALLRPGRFDRQVSVDLPDQKGRLEILKVHARNKKVAEDVDLQEVAMRTPGFA  421
SynFtsH1      337 LLTEMDGFEGNSGIIVIAATNRPDVLDLALLRPGRFDRQVTVDYPDVQGRELILAIHAQNKKLHEEVQLAAIARRTPGFT  416
SynFtsH2      298 LLTEMDGFEGNTGIIIIAATNRPDVLDSALMRPGRFDRQVMVDAPDYSGRKEILEVHARNKKLAPEVSIDSIARRTPGFS  377
                  .*******.**.*.*. *****.*.**.**.********* ** **.  **  **..*...*..   .*..  .*.*****.

                                                                       Zn-binding
AtFtsH1       468 GADLQNLMNEAAILAARRELKEISKDEISDALERIIAGPEKKNAVVSEEKKRLVAYHEAGHALVGALMPEYDPVAKISII  547
AtFtsH5 (VAR1)456 GADLQNLMNEAAILAARRELKEISKDEISDALERIIAGPEKKNAVVSEEKKRLVAYHEAGHALVGALMPEYDPVAKISII  535
CrFtsH1       466 GADLQNLMNEAAILAARRNLKEISKEEIADALERIIAGPEKKGAVMSDKKRRLVAYHEAGHALVGALMPEYDPVTKISIV  545
SynFtsH3      367 GADLSNLLNEAAILAARRNLTEISMDEVNDAIDRVLAGPEKKNRVMSEKRKTLVAYHEAGHALVGALMPDYDPVQKISII  446
AtFtsH2 (VAR2)433 GADLANLLNEAAILAGRRARTSISSKEIDDSIDRIVAGMEGT-VMTDGKSKSLVAYHEVGHAVCGTLTPGHDAVQKVTLI  511
AtFtsH8       426 GADLANLLNEAAILAGRRGKTAISSKEIDDSIDRIVAGMEGT-VMTDGKSKSLVAYHEVGHAICGTLTPGHDAVQKVTLI  504
CrFtsH2       422 GANLMNLLNEAAILAGRRGLKAITNKEIDDAIDRIVAGLEGK-PLVDGKAKALVAYHEVGHAICGTLQPGHDPVQKVTLV  500
SynFtsH1      417 GADLANVLNEAAIFTARRRKEAITMAEVNDAIDRVVAGMEGT-PLVDSKSKRLIAYHEVGHALIGTLCPGHDPVEKVTLI  495
SynFtsH2      378 GADLANLLNEAAILTARRRKSAITLLEIDDAVDRVVAGMEGT-PLVDSKSKRLIAYHEVGHAIVGTLLKDHDPVQKVTLI  456
                  **.* *..*****...**  .  *.  *. *...*..** *..    .. . ..*.****.***..*.*  . .*.*.*....

AtFtsH1       548 PRGQAGGLTFFAPSEERLESGLYSRSYLENQMAVALGGRVAEEVIFGDENVTTGASNDFMQVSRVARQMIERFGFSKKIG  627
AtFtsH5 (VAR1)536 PRGQAGGLTFFAPSEERLESGLYSRSYLENQMAVALGGRVAEEVIFGDENVTTGASNDFMQVSRVARQMVERFGFSKKIG  615
CrFtsH1       546 PRGAAGGLTFFAPSEERLESGLYSRTYLENQMAVALGGRIAEELIFGEDDITTGASGDFQQVTRIARLMVTQLGLSKKLG  625
SynFtsH3      447 PRGRAGGLTWFTPSEDRMESGLYSRSYLQNQMAVALGGRIAEEIIFGEEEVTTGASNDLQQVARVARQMVTRFGMSDRLG  526
AtFtsH2 (VAR2)512 PRGQARGLTWFIPSDDP---TLISKQQLFARIVGGLGGRAAEEIIFGDSEVTTGAVGDLQQITGLARQMVTTFGMSD-IG  587
AtFtsH8       505 PRGQARGLTWFIPSDDP---TLISKQQLFARIVGGLGGRAAEEVIFGESEVTTGAVSDLQQITGLAKQMVTTFGMSE-IG  580
CrFtsH2       501 PRGQARGLTWFIPGEDP---TLVSKSQIFARIVGALGGRAAEELVFGEDEVTSGAASDLQQVSGMARQMVINYGMSN-IG  576
SynFtsH1      496 PRGQAQGLTWFTPDEDQ---SLMTRNQMIARIAGLLGGRVAEEVIFGDDEVTTGAGNDIEKITYLARQMVTKLGMSS-LG  571
SynFtsH2      457 PRGQAQGLTWFTPNEEQ---GLTTKAQLMARIAGAMGGRAAEEEVFGDDEVTTGAGGDLQQVTEMARQMVTRFGMSN-LG  532
                  ***.* ***.* *...     .*  .. .. .......*** ***  .**. ..*.**  *.....   *..*.. .*.*   .*

AtFtsH1       628 QVAVGGPGGNP-FMGQQMSSQKDYSMATADIVDAEVRELVEKAYKRATEIITTHIDILHKLAQLLIEKETVDGEEFMSLF  706
AtFtsH5 (VAR1)616 QVAVGGAGGNP-FLGQSMSSQKDYSMATADVVDAEVRELVEKAYVRAKEIITTQIDILHKLAQLLIEKETVDGEEFMSLF  694
CrFtsH1       626 QVAWSNQGGAS-FLGASAAQPADFSQSTADEIDSEVKELVERAYRRAKDLVEQNIDILHKVAAVLIEKENIDGDEFQQIV  704
SynFtsH3      527 PVALGRQGGGV-FLGRDIASDRDFSDETAAAIDEEVSQLVDQAYQRAKQVLVENRGILDQLAEILVEKETVDSEELQTLL  605
AtFtsH2 (VAR2)588 PWSLMDSSAQS-DVIMRMMARNSMSEKLAEDIDSAVKKLSDSAYEIALSHIKNNREAMDKLVEVLLEKETIGGDEFRAIL  666
AtFtsH8       581 PWSLMDSSEQS-DVIMRMMARNSMSEKLANDIDTAVKTLSDKAYEIALSQIRNNREAMDKIVEILLEKETMSGDEFRAIL  659
CrFtsH2       577 PWSLMDPSAMSGDMIMRMMSRNSMSESLQQRIDSQVRTIADQAYEVALRHIADNREAIDRIVEALMEKETLTGDEFRAML  656
SynFtsH1      572 LVALEEEGDRN-FSGGDWGKRSEYSEDIAARIDREIQAIVTAAHQRATRIIEENRNLMDLLVDALIDQETIEGEHFRQLV  650
SynFtsH2      533 PISLESSGGEV-FLGGGLMNRSEYSEEVATRIDAQVRQLAEQGHQMARKIVQEQREVVDRLVDLLIEKETIDGEEFRQIV  611
                  ....   ..   . .  .   .    *.  .  .* ..  ..  .. .*   .  ..   ....  *...*. ......

AtFtsH1       707 IDGQAELYIS-----------------------                                                716
AtFtsH5 (VAR1)695 IDGQAELYVS-----------------------                                                704
CrFtsH1       705 LASQAQQYTKKDAPGVSIPYQAA----------                                                727
SynFtsH3      606 ANNNAKLALLV----------------------                                                616
AtFtsH2 (VAR2)667 SEFTEIPPENRVPSSTTTTPASAPTPAAV----                                                695
AtFtsH8       660 SEFTEIPPENRVASSTSTSTPTPASV-------                                                685
CrFtsH2       657 AEYTTIPEENVKAVEAQRKGGAELVAAATRMEL                                                689
SynFtsH1      651 ESYQQSQKQPALAGK------------------                                                665
SynFtsH2      612 AEYAEVPVKEQLIPQL-----------------                                                627
```

Supplementary FIGURE 4. Localization of phosphorylated residues in FtsH proteins. Amino acid sequence of FtsH proteins in *Arabidopsis thaliana*, *Chlamydomonas reinhardtii*, and *Synechocystis* sp. PCC 6803 were aligned. The phosphorylation sites which experimentally determined by phosphoproteome in *Arabidopsis thaliana* and *Chlamydomonas reinhardtii* are highlighted in red. Conserved domains of ATPase family members, including Walker A and B motifs and the second region of homology (SRH) are in yellow. The zinc binding motif (Zn-binding) is shown in green. Conserved consensus sequence AXA that is recognized by the thylakoid processing peptidase (TPP) is highlighted in pink. The transmembrane domain (TM) is highlighted in gray.

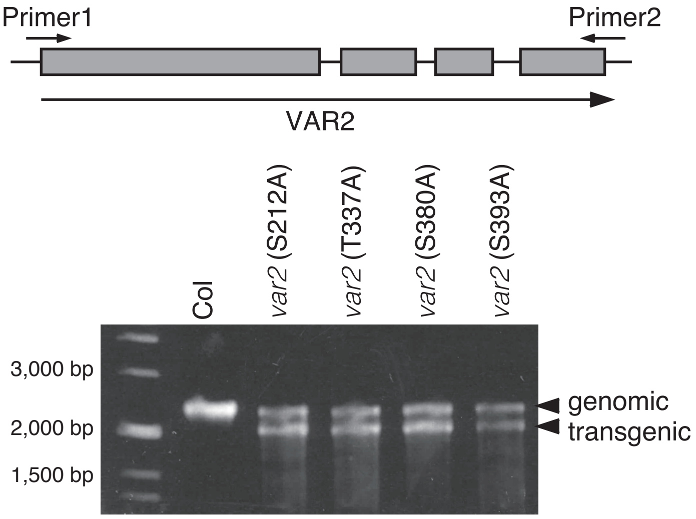

Supplementary FIGURE 5. The genotyping of transgenic lines. Primer sets used for genotyping are shown in Supplementary Table S1. The upper panel showed the primer position. The upper band is PCR product of genomic *VAR2* and the lower band is PCR product of transgene. In *var2-1* allele, the stop codon mutation still allows PCR amplification of genomic *VAR2*.
